# COVID-19 and Vaccine Hesitancy: Individual Determinants Among Saudis in Asir Region

**DOI:** 10.7759/cureus.22331

**Published:** 2022-02-17

**Authors:** Muneer Jan, Mushary Alqahtani, Khaled A Amer, Basel Althubait, Abdulrahman Ali S Aldosari, Abdulrahman Abdullah M Al mudawi

**Affiliations:** 1 Department of Surgery, King Khalid University, Abha, SAU; 2 College of Medicine, King Khalid University, Abha, SAU

**Keywords:** vaccine hesitancy, hesitancy, saudi arabia, public, intent, covid-19 vaccine

## Abstract

Background

Coronavirus disease 2019 (COVID-19) is a highly contagious and quickly spreading disease, especially if associated with poor awareness and unwanted behavioral practices. Unvaccinated people are at high risk of infection, mortality, and morbidity. Practices and intent toward the COVID-19 vaccine are mainly influenced by the perception of vaccine safety. This study aimed to assess the perception, practices, intent, and challenges toward the COVID-19 vaccine in Asir region, Saudi Arabia.

Methodology

A quantitative, cross-sectional study was conducted among the available population in Asir region, southwest of Saudi Arabia. Data were collected from participants using a semi-structured electronic questionnaire. The questionnaire included sections on participants’ socio-demographic data and their family and personal history of COVID-19 infection. Additionally, the effects of the COVID-19 pandemic on their daily life were assessed. The second section of the questionnaire included knowledge questions regarding the COVID-19 vaccine with only one correct answer for each question. The last section covered participants’ attitudes toward the COVID-19 vaccine and their intent to take the vaccine with their previous vaccination practice.

Results

A total of 756 participants who met the inclusion criteria participated in the study. Participants’ ages ranged from 18 to 65 years, with a mean age of 22.6 ± 12.8 years. A total of 518 respondents were females. Regarding the level of education, 72.2% were university graduates or postgraduates, and 195 (25.8%) were in high school. Regarding the overall knowledge level toward the COVID-19 vaccine among study participants, 420 (55.6%) participants had good knowledge regarding the COVID-19 vaccine. In total, 158 (20.9%) participants were of the view that the COVID-19 vaccine is risky. Approximately 26.9% of the participants were told by their doctor that vaccination is necessary, and 49.3% thought that they need more information about the COVID-19 vaccine.

Conclusions

This study revealed that public awareness regarding the COVID-19 vaccine was satisfactory, especially concerning its benefit in reducing infection and associated complications; however, poor awareness was reported regarding their perception of the pandemic and COVID-19 vaccine definition.

## Introduction

Coronavirus disease 2019 (COVID-19), caused by the severe acute respiratory syndrome coronavirus 2 (SARS‐CoV‐2), is a highly transmissible and quickly spreading disease [1]. The COVID-19 outbreak started in Wuhan, Hubei province, China, in December 2019, and by early 2020, the world was facing a rapidly spreading epidemic, which was later classified by the World Health Organization (WHO) as a pandemic [2]. WHO classified the disease as a Public Health Emergency of International Concern on January 30, 2020, and recognized it as a pandemic on March 11, 2020 [3,4].

Considerable efforts were made toward the development of vaccines against COVID-19 to prevent the pandemic. The majority of the emerging vaccines have used the S-protein of SARS-CoV-2 [5]. The most efficient approach to control the spread of the COVID-19 pandemic, in the long run, is mass vaccination [6]. The efficacy of the COVID-19 vaccination is mainly based on continuous vaccination, and its preparation and delivery inventiveness. In November 2020, several international pharmaceutical companies reported the effectiveness and benefit of their vaccines according to large clinical trials [7]. Because of the urgent situation caused by the pandemic, these vaccines received emergency regulatory approvals by national drug and pharmaceutical agencies in December 2020, and vaccination programs were rolled out in many countries soon after [8,9].

Owing to poor knowledge, attitude, and practices toward preventing COVID-19 among communities in Saudi Arabia, the absence of vaccines or treatments can lead to further disease transmission and place these communities at higher risk of infection, mortality, and morbidity [10]. Recently, COVID-19 vaccines have become available in Saudi Arabia, which is a significant preventative measure against virus transmission. However, it is important to determine the knowledge, attitudes, practices, and intent of individuals toward getting the COVID-19 vaccine, which are mainly influenced by their knowledge level, attitudes, practices, and intent [11]. This study aimed to assess the perception, practices, intent, and challenges toward the COVID-19 vaccine in the Asir region of Saudi Arabia.

## Materials and methods

A quantitative, cross-sectional survey was conducted among the available population in the Asir region, Saudi Arabia. This study was approved by the Research Ethics Committee at King Khalid University (Approval number: ECM#2021-5307). Individuals aged 18 years or more living in the Asir region for at least six months were invited to voluntarily participate in the survey. A total of 1,200 eligible individuals were included in the survey. In total, 756 participants completed the study questionnaire, with a response rate of 63.8%. Data were collected from participants using a semi-structured electronic questionnaire. Questionnaire items were reviewed by a panel of three experts from the College of Medicine at King Khalid University to check their applicability and content validity. Tool reliability was assessed using a pilot study of 25 participants, with a reliability coefficient (Cronbach’s alpha) of 0.71. The questionnaire included the following items: participants’ socio-demographic data, such as age, gender, education, income, and employment status, their family and personal history of COVID-19 infection, and the effect of the COVID-19 pandemic on their daily life. The second section included knowledge questions regarding the COVID-19 vaccine, with only one correct answer for each question. The final section covered participants’ attitudes toward the COVID-19 vaccine and their intent to take the vaccine with their previous vaccination practice. The questionnaire was uploaded online using social media platforms by researchers and their friends, and all eligible individuals were invited to fill it after explaining the study purpose and assuring them of data confidentiality.

Data analysis

After extraction, data were revised, coded, and analyzed using SPSS version 22 (IBM Corp., Armonk, NY, USA). All statistical analyses were done using two-tailed tests. P-values less than 0.05 were considered statistically significant. For knowledge and awareness items, each correct answer was scored one point, and the total sum of the discrete scores of the different items was calculated. A patient with a score less than 60% (0-3 points) of the total score was considered to have poor awareness, while good awareness was considered if the score was 60% (4 points or more) of the total or more. Descriptive analysis based on the frequency and percentage distribution was performed for all variables, including participants’ socio-demographic data, family and personal history of COVID-19 infection, the risk level for being infected with COVID-19, and the impact of the pandemic on their life. Moreover, participants’ knowledge and awareness regarding the COVID-19 vaccine, attitude, and intent to get the vaccine were presented as frequency tables and graphs. Crosstabulation was used to assess the distribution of public knowledge and the awareness level regarding the COVID-19 vaccine according to their personal data, disease history, and family history. Furthermore, the relationship between participants’ knowledge level and their attitude with intent to have the vaccine was tabulated. Relationships were determined using Pearson’s chi-square test and exact probability test for small frequency distributions.

## Results

A total of 756 participants who met the inclusion criteria participated in the study. Participants’ ages ranged from 18 to 65 years, with a mean age of 22.6 ± 12.8 years. In total, 518 respondents were females. Regarding the level of education, 72.2% were university graduates or postgraduates, and 195 (25.8%) were in high school. Of the total study population, 435 (57.5%) were students, 146 (19.3%) were not employed, and 23.1% were employed in the government or private sector. In total, 98 (13%) participants had chronic health problems, including chronic respiratory disease (33.8%), diabetes (14.3%), obesity (6%), and hypertension (4.5%), while 3% had autoimmune health problems. A total of 464 (61.4%) participants had a history of COVID-19 infection among their family and friends. Moreover, 25.9% reported that they were at very high to high risk for contracting COVID-19 infection in the coming months, while 28.1% reported being at low to very low risk. Regarding the impact of the pandemic on daily life, large to extremely large effect was reported by 65.6% of the study participants, while only 8.2% reported low impact (Table 1).

**Table 1:** Socio-demographic data of study participants. COVID-19: coronavirus disease 2019

Socio-demographic data		No.	%
Age (years)	18–25	515	68.1%
	26–30	83	11.0%
	31–40	85	11.2%
	41–50	43	5.7%
	51+	30	4.0%
Gender	Male	238	31.5%
	Female	518	68.5%
Highest level of education	Middle school/below	15	2.0%
	High school	195	25.8%
	University graduate	497	65.7%
	Postgraduate degree	49	6.5%
Marital status	Single	574	75.9%
	Married	170	22.5%
	Divorced/Widowed	12	1.6%
Employment	Unemployed/Retired	146	19.3%
	Student	435	57.5%
	Governmental employee	94	12.4%
	Private sector employee	81	10.7%
Chronic diseases	Yes	98	13.0%
	No	658	87.0%
Type of the disease	Respiratory chronic diseases	45	33.8%
	Diabetes	19	14.3%
	Obesity	8	6.0%
	Hypertension	6	4.5%
	Autoimmune diseases	4	3.0%
	Cardiac diseases	3	2.3%
	Other	47	35.3%
History of COVID-19 infection in family and friends	Yes	464	61.4%
	No	292	38.6%
What is your level of risk for COVID-19 infection in the coming months	Very high	72	9.5%
	High	124	16.4%
	Average	348	46.0%
	Low	145	19.2%
	Very low	67	8.9%
Impact of the pandemic on daily life	Extremely large	402	53.2%
	Large	94	12.4%
	Fair	198	26.2%
	Small	15	2.0%
	Extremely small	47	6.2%

As shown in Table 2, 16.9% of the participants correctly defined COVID-19 infection, while 22.5% knew about the COVID-19 vaccine. Additionally, 57.5% of the respondents agreed that the vaccine will reduce/prevent the risk of infection, and 80.7% knew that the COVID-19 vaccine was beneficial. Further, 53.2% agreed that taking the COVID-19 vaccine can lower the risk of complications. The most reported source of information regarding the COVID-19 vaccine was social media (48.8%), followed by healthcare staff (13.1%), internet (11.5%), and others (5%).

**Table 2:** Participants’ knowledge regarding the COVID-19 vaccine. COVID-19: coronavirus disease 2019

Knowledge items	No	%
What is COVID-19 infection?		
Incorrect definition	128	16.9%
Correct definition	628	83.1%
What is vaccination?		
Incorrect definition	170	22.5%
Correct definition	586	77.5%
The vaccine will reduce/prevent the risk the infection		
Agree	435	57.5%
Maybe	230	30.4%
Disagree	53	7.0%
I don’t know	38	5.0%
Is COVID-19 vaccination beneficial?		
Yes	610	80.7%
No	146	19.3%
Source of information		
Social media	369	48.8%
General physician or family physician	99	13.1%
Internet	87	11.5%
Other	38	5.0%
Family	20	2.6%
Friends	10	1.3%
None	133	17.6%
Taking the COVID-19 vaccine lowers complications		
Agree	402	53.2%
Maybe	246	32.5%
Disagree	67	8.9%
I don’t know	41	5.4%

**Figure 1:**
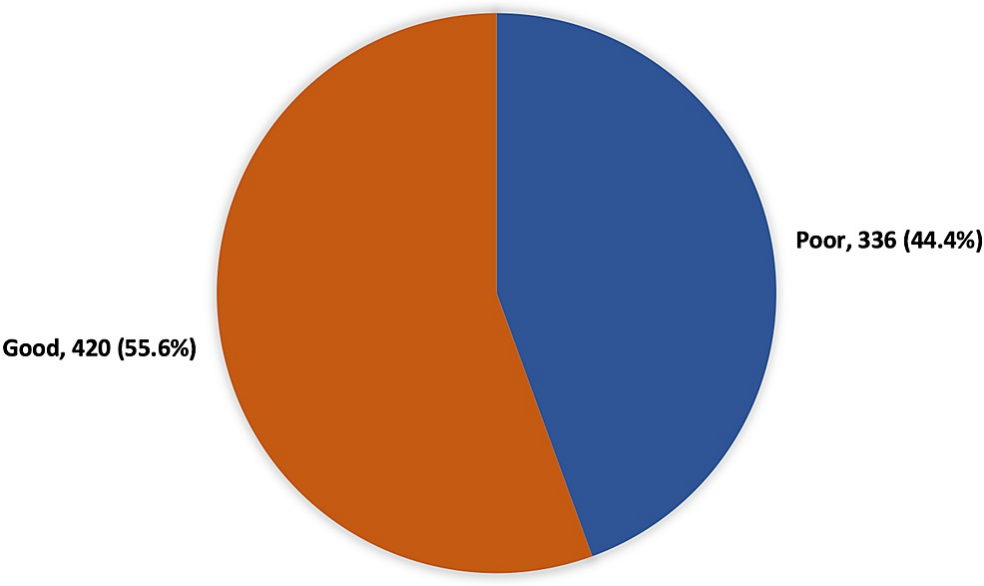
The overall knowledge level regarding the COVID-19 vaccine among study participants. COVID-19: coronavirus disease 2019

Figure 1 shows that 420 (55.6%) participants had a good knowledge level regarding the COVID-19 vaccine while 336 (44.4%) had a poor knowledge level.

Table 3 demonstrates that 158 (20.9%) participants thought that the COVID-19 vaccine was risky. The most reported sources regarding the vaccine risk were social media (40.5%), the internet (18.4%), and family and friends (17.1%). On the other hand, 26.9% of the participants were told by their doctor that vaccination was necessary, and 49.3% thought that they needed more information about the vaccine.

**Table 3:** Participants’ attitude and perception toward the COVID-19 vaccine. COVID-19: coronavirus disease 2019

Attitude and perception	No	%
Do you think the vaccine is risky?		
Yes	158	20.9%
No	598	79.1%
Source of risk information		
Social media	64	40.5%
Other	38	24.1%
Internet	29	18.4%
Family	16	10.1%
Friends	11	7.0%
Have you ever been told by your doctor that vaccination is absolutely necessary?		
Yes	203	26.9%
No	92	12.2%
I didn’t ask about it	461	61.0%
Do you need more information about the COVID-19 vaccine?		
Yes	373	49.3%
No	383	50.7%

As presented in Table 4, 24.3% of the participants reported they could change their mind about agreeing or refusing to take the vaccine while 58.6% may change their mind. Moreover, 77.9% of the participants reported that they will accept taking the vaccine themselves or for their family, and 78.7% told if a lot of people had the vaccine, they will take it. In total, 116 (15.3%) participants reported that they had refused vaccination of a certain vaccine in the past.

**Table 4:** Participants’ intent and practice regarding the COVID-19 vaccine. COVID-19: coronavirus disease 2019

Vaccine intent and practice	No	%
Do you think you can change your mind about accepting or refusing to take the vaccine?		
Yes	184	24.3%
Maybe	445	58.9%
No	127	16.8%
If you were asked to take the vaccine for yourself and your family, will you accept it?		
Yes	589	77.9%
Maybe	112	14.8%
No	55	7.3%
If a lot of people had the vaccine, are you going to take it?		
Yes	595	78.7%
May be	98	13.0%
No	63	8.3%
Did you refuse vaccination of a certain type of vaccine in the past?		
Yes	116	15.3%
No	640	84.7%

Table 5 shows that a good knowledge level was detected among 62.2% of male participants versus 52.5% of female participants, which was statistically significant (P = 0.013). Further, 62.8% of governmental employees had good knowledge regarding the vaccine compared to 44.4% of those employed in the private sector and 47.9% of the unemployed group (P = 0.011). Good knowledge level was detected among 59.2% of those with an average level of risk for COVID-19 infection compared to 41.8% of those with a low level of risk (P = 0.049).

**Table 5:** Distribution of participants’ knowledge level regarding the COVID-19 vaccine according to their socio-demographic data. P: Pearson’s chi-square test; $: exact probability test; *: P < 0.05 (significant). COVID-19: coronavirus disease 2019

Factors	Knowledge level				P-value
	Poor		Good		
	No	%	No	%	
Age (years)					0.664
18–25	222	43.1%	293	56.9%	
26–30	38	45.8%	45	54.2%	
31–40	39	45.9%	46	54.1%	
41–50	20	46.5%	23	53.5%	
51+	17	56.7%	13	43.3%	
Gender					0.013*
Male	90	37.8%	148	62.2%	
Female	246	47.5%	272	52.5%	
Highest level of education					0.838^$^
Middle school/below	5	33.3%	10	66.7%	
High school	88	45.1%	107	54.9%	
University graduates	222	44.7%	275	55.3%	
Postgraduate degree	21	42.9%	28	57.1%	
Employment					0.011*
Unemployed/Retired	76	52.1%	70	47.9%	
Student	180	41.4%	255	58.6%	
Governmental employee	35	37.2%	59	62.8%	
Private sector employee	45	55.6%	36	44.4%	
Had chronic diseases					0.333
Yes	48	49.0%	50	51.0%	
No	288	43.8%	370	56.2%	
History of COVID-19 infection in family and friends					0.124
Yes	196	42.2%	268	57.8%	
No	140	47.9%	152	52.1%	
What is your level of risk for COVID-19 infection in the coming months					0.049*
Very high	34	47.2%	38	52.8%	
High	60	48.4%	64	51.6%	
Average	142	40.8%	206	59.2%	
Low	61	42.1%	84	57.9%	
Very low	39	58.2%	28	41.8%	
Impact of the pandemic on daily life					0.103
Extremely large	169	42.0%	233	58.0%	
Large	35	37.2%	59	62.8%	
Fair	99	50.0%	99	50.0%	
Small	9	60.0%	6	40.0%	
Extremely small	24	51.1%	23	48.9%	

Table 6 shows that a higher knowledge level was significantly associated with a lower attitude about the vaccine risk. Overall, 93.1% of those with a good knowledge level accepted to get the vaccine compared to 58.9% of those with poor knowledge (P = 0.001). In addition, 92.6% of participants with good knowledge regarding the vaccine agreed to take the vaccine if many people had it versus 61.3% of those with poor knowledge level (P = 0.001). On the other hand, changing their mind regarding vaccine acceptance was significantly higher among those with poor knowledge levels compared to those with higher knowledge (29.5% vs. 20.2%, respectively).

**Table 6:** Distribution of participants’ attitude and intent toward the COVID-19 vaccine according to their knowledge level. P: Pearson’s chi-square test; $: exact probability test; *: P < 0.05 (significant). COVID-19: coronavirus disease 2019

Vaccine attitude and intent	Knowledge level				P-value
	Poor		Good		
	No	%	No	%	
Do you think the vaccine is risky?					0.001*
Yes	116	34.5%	42	10.0%	
No	220	65.5%	378	90.0%	
Have you ever been told by your doctor that vaccination is absolutely necessary?					0.001*
Yes	67	19.9%	136	32.4%	
No	44	13.1%	48	11.4%	
I didn’t ask about it	225	67.0%	236	56.2%	
Do you need more information about the COVID-19 vaccine?					0.026*
Yes	181	53.9%	192	45.7%	
No	155	46.1%	228	54.3%	
Do you think you can change your mind about accepting or refusing to take the vaccine?					0.001*
Yes	99	29.5%	85	20.2%	
Maybe	198	58.9%	247	58.8%	
No	39	11.6%	88	21.0%	
If you were asked to take the vaccine for yourself and your family, will you accept it?					0.001*
Yes	198	58.9%	391	93.1%	
Maybe	89	26.5%	23	5.5%	
No	49	14.6%	6	1.4%	
If a lot of people had the vaccine, are you going to take it?					0.001*^$^
Yes	206	61.3%	389	92.6%	
Maybe	73	21.7%	25	6.0%	
No	57	17.0%	6	1.4%	

## Discussion

The public unwillingness to receive safe and suggested available vaccines, known as “vaccine hesitancy,” was already a rising issue before the COVID-19 pandemic [12]. An outline established from research in high-income countries, namely, “the 5C model of the drivers of vaccine hesitancy,” describes the following five main person-level factors for vaccine hesitancy: confidence, complacency, convenience (or constraints), risk calculation, and collective responsibility [13,14]. Encouraging the intake of vaccines, especially those against COVID-19, requires understanding whether people are willing to be vaccinated, the motives why they are ready or not ready to do so, the most trusted sources of information in their decision-making, and their awareness levels. Wouters et al. assessed these factors using a public set of surveys conducted between June 2020 and January 2021 in 15 studies carried out in Africa, South Asia, Latin America, Russia, and the United States [15].

Health knowledge establishes a solid contextual factor that endorses health prevention activities. Higher knowledge levels about health risks, signs and symptoms, and the benefits of preventive actions promote healthier lifestyles [16]. The knowledge, attitude, and behavior assume that a person’s health awareness and information play an important role in health-related behavior [17].

This study aimed to assess the public knowledge level, attitude, and intent toward the COVID-19 vaccine and their effect on the willingness to get the vaccine. Regarding public knowledge and awareness, the findings showed that more than half of the participants had a good knowledge level regarding the COVID-19 vaccine. More than half of the participants (57.5%) knew that the vaccine will reduce/prevent the risk of the infection, while a vast majority of the participants (80.7%) knew that the COVID-19 vaccine is beneficial. Furthermore, more than half agreed that taking the COVID-19 vaccine will reduce the risk of complications. On the other hand, lower awareness regarding the virus and vaccine was reported, with less than one-fifth of the respondents correctly defining COVID-19 infection while about one-fifth (22.5%) knew about the COVID-19 vaccine. Additionally, the most reported source of information regarding the COVID-19 vaccine was social media (48.8%), followed by healthcare staff (13.1%), the internet (11.5%), and others (5%). A higher awareness level was reported by Elgendy et al [18], where the median score of the survey was 20/22 regarding knowledge about the COVID-19 vaccine. Overall, the study participants had good knowledge about the COVID-19 vaccine and accepted to take the vaccine, indicating the highly commendable efforts to control the coronavirus. In Saudi Arabia, Alrefaei et al. [19] found that all respondents knew that the coronavirus is contagious, and 89.8% agreed that the symptoms of COVID-19 are similar to those of the seasonal flu. A high level of knowledge of the main factors of SARS-CoV-2 transmission was also reported. More than 98.7% of respondents knew about the role of large gatherings and events in the further spread of the virus. However, the respondents considered COVID-19 vaccines to be effective, but some respondents were not aware of their side effects, and 38.8% planned to receive a vaccine. Similar findings were reported among participants in Riyadh [20].

Regarding participants’ attitudes and intent toward the COVID-19 vaccine, the findings showed that one-fifth of the participants thought that the COVID-19 vaccine is risky. Moreover, only about one-fourth of the participants were told by their doctor that vaccination is necessary, which explains that about half of the respondents thought that they need more information about the COVID-19 vaccine. Additionally, about one out of four participants reported possibly changing their mind about accepting or refusing to take the vaccine, while more than half (58.6%) may change their opinion. Nearly three-quarters of the respondents reported that they will accept the vaccine for themselves or for their family, reported if a lot of people had the vaccine, they will take it. Similar findings were reported by Al-Zalfawi et al. [21] as the majority of respondents (76%) had satisfactory knowledge, a positive attitude (72.4%), and perception (71.3%) toward the use of COVID-19 vaccines. In Jazan, Almalki [22] reported that participants demonstrated a good knowledge of COVID-19, correctly answering 77% of the knowledge questions. Most of the participants exhibited good attitudes and acceptable practices toward COVID-19.

Limitations and strengths

Even though this study is one of the few studies in the region identifying the challenges associated with COVID-19 vaccine hesitancy, the study had some limitations. First, as the data were collected through a self-reported method, recall bias and question misinterpretation could have occurred. Further, we relied on online platforms as a primary tool to collect the data, which might explain the gender differences in our sample. In addition, we conducted this study in the Asir region of Saudi Arabia, which might affect its generalizability.

## Conclusions

This study revealed that public awareness regarding the COVID-19 vaccine was satisfactory, especially regarding its benefit in reducing infection and associated complications; however, poor awareness was reported regarding their perception of the pandemic and COVID-19 vaccine definition. Furthermore, participants’ attitudes and intent to have the vaccine were high, especially among those with high knowledge levels. The study showed that the role of the healthcare staff in providing information regarding the pandemic and vaccine efficacy was not satisfactory, with more effort needed to improve their awareness and perception regarding the vaccine. Providing adequate information about the vaccines is recommended. Continuous training and education are needed to improve public vaccine acceptance and reduce vaccine hesitancy.
